# Supplementary material for: SPF45/RBM17-dependent, but not U2AF-dependent, splicing in a distinct subset of human short introns
Source: Nat Commun. 2021 Aug 13;12:4910. doi: 10.1038/s41467-021-24879-y (PMC8363638; doi:10.1038/s41467-021-24879-y)
Supplement: Supplementary file 1 — Supplementary File [file 41467_2021_24879_MOESM1_ESM.pdf]

SPF45/RBM17-dependent, but not U2AF-dependent, splicing in a distinct subset of human short introns

Kazuhiro Fukumura[1]*, Rei Yoshimoto[1], Luca Sperotto[2,3], Hyun-Seo Kang[2,3], Tetsuro Hirose[4], Kunio Inoue[5], Michael Sattler[2,3] & Akila Mayeda[1]*

[1]Division of Gene Expression Mechanism, Institute for Comprehensive Medical Science, Fujita Health University, Toyoake, Aichi 470-1192, Japan
[2]Institute of Structural Biology, Helmholtz Zentrum München, 85764 Neuherberg, Germany
[3]Biomolecular NMR and Center for Integrated Protein Science Munich, Chemistry Department, Technical University of Munich, 85748 Garching, Germany
[4]Institute for Genetic Medicine, Hokkaido University, Sapporo, Hokkaido 060-0815, Japan
[5]Department of Biology, Graduate School of Science, Kobe University, Kobe, Hyogo 657-8501, Japan

*e-mails: fukumura@fujita-hu.ac.jp (K.F.), mayeda@fujita-hu.ac.jp (A.M.)

Contents

Table S1
Table S2
Fig. S1
Fig. S2
Fig. S3
Fig. S4
Fig. S5
Fig. S6
Fig. S7
Fig. S8
Fig. S9

Data 1
(Uploaded in a separate Excel file)

Table S1 List of siRNA library targeting human nuclear proteins and the results of each knockdown
*PSI (Percent Spliced In) in this Table is the ratio of retained intron inclusion (Percent Retained-intron In)

No	Protein/gene name (HGNC gene symbol)	Knockdown (KD) efficiency (Control KD=1)	PSI* (control KD=0.103)
1	Acinus (ACIN1)	0.580	0.149
2	AHDC1	0.007	0.264
3	AKAP149 (AKAP1)	0.307	0.231
4	AKAP8L	0.042	0.233
5	Aly C12 (ALYREF)	0.075	0.107
6	ASR2B (SRRT)	0.018	0.134
7	Ataxin1 (ATXN1)	Undetectable	N/A
8	Barentsz (CASC3)	0.120	0.116
9	CArG-binding factor A (NFYB)	0.404	0.149
10	CBP20 (NCBP2)	0.049	0.094
11	CBX7	Undetectable	N/A
12	CELF5	Undetectable	N/A
13	CELF6	Undet.	N/A
14	Cflm68 (CPSF6)	0.160	0.111
15	CIRBP	0.468	0.159
16	CLK1	0.746	0.113
17	CLK4	0.154	0.127
18	CPSF5 (NUDT21)	0.026	0.180
19	CPSF7	0.109	0.220
20	CUGBP (CELF1)	0.039	0.195
21	DAZAP1	Undetectable	N/A
22	DGCR8	1.220	N/A
23	ECM2	Undetectable	N/A
24	ELAVlike (ELAVL1)	0.039	0.102
25	ES349 (FUBP1)	0.276	0.137
26	EWS (EWSR1)	0.072	0.226
27	F9/11A1 (DDX18)	0.034	0.202
28	F9/17A4 (NUP153)	0.079	0.134
29	F9/29D5 (SRM160)	0.267	0.269
30	F9/3B6 (ZNF346)	0.329	0.102
31	FAM113A (PCED1A)	0.031	0.137
32	FAM98A	0.062	0.195
33	FIGN	Trace	0.211
34	FLJ10005 (SLTM)	0.227	0.152
35	FLJ10968 (IM3)	0.026	0.176
36	FLJ20273 (RBM47)	Undetectable	N/A
37	FUBP3	0.125	0.204
38	FUS	0.117	0.249
39	FXR1	0.076	0.172
40	FXR2	0.107	0.240
41	GRSF1	0.026	0.172
42	GRY-RBP (SYNCRIP)	0.034	0.151
43	hnRNP A/B (HNRNPAB)	0.035	0.143
44	hnRNP D-like (HNRNPDLP1)	Undetectable	N/A
45	hnRNP RALY (RALY)	0.016	0.202
46	hnRNP A0 (HNRNPA0)	0.595	0.202
47	hnRNP A1 (HNRNPA1)	0.045	0.102
48	hnRNP A2/B1 (HNRNPA2B1)	0.019	0.162
49	hnRNP A3 (HNRNPA3)	0.101	0.130
50	hnRNPC1/C2 (HNRNPC)	0.025	0.104
51	hnRNP D (HNRNPD)	0.007	0.132
52	hnRNP E1 (PCBP1)	0.027	0.254
53	hnRNP F (HNRNPF)	0.061	0.114
54	hnRNP H1 (HNRNPH1)	0.104	0.393
55	hnRNP H2 (HNRNPH2)	0.153	0.148
56	hnRNP H3 (HNRNPH3)	0.022	0.158
57	hnRNP K (HNRNPK)	0.022	0.218
58	hnRNP L (HNRNPL)	0.004	0.203
59	hnRNP M (HNRNPM)	0.006	0.196
60	hnRNP R (HNRNPR)	0.082	0.242
61	hnRNP U-like 1 (HNRNPUL1)	0.045	0.125
62	HP1a (CBX5)	0.104	0.206
63	IMP1 (HM13)	0.805	0.176
64	IMP3	0.369	0.102
65	kin 17 (KIN)	0.188	0.164
66	KSRP (KHSRP)	0.101	0.201
67	La atoantigen (SSB)	0.451	0.160
68	Matrin3 (MATR3)	0.430	0.169
69	MBD1	0.061	0.075
70	MBD2	0.108	0.202
71	MBNL (MBNL1)	0.109	0.127
72	MECP2	0.114	0.190
73	MEX3A	Undetectable	N/A
74	MEX3B	Undetectable	N/A
75	MEX3D	Undetectable	N/A
76	MINT (SPEN)	3.404	N/A
77	MSP58 (MCRS1)	0.018	0.267
78	MSY2 (YBX2)	Undetectable	N/A

No	Protein/gene name (HGNC gene symbol)	Knockdown (KD) efficiency (Control KD=1)	PSI (control KD=0.103)
79	NOL8	0.291	0.151
80	Nopp34 (NIFK)	0.341	0.179
81	Nucleolin (NCL)	0.691	0.304
82	Peptidylprolyl isomerase E (PPIE)	0.015	0.135
83	POLDIP3	0.006	0.146
84	PPARGC1 (PPARGC1A)	0.039	0.252
85	PSF (SFPQ)	Undetectable	N/A
86	PSP1 (PSPC1)	1.335	N/A
87	PSP2 (RBM14)	0.016	0.074
88	PTB (PTBP1)	0.009	0.173
89	PUF60	0.009	0.267
90	Puralpha (PURA)	0.076	0.196
91	p54nrb (NONO)	0.029	0.102
92	RAVER1	Undetectable	N/A
93	RBM10	0.031	0.239
94	RBM12	0.254	0.140
95	RBM15	0.387	0.248
96	RBM19	0.033	0.412
97	RBM22	0.012	0.241
98	RBM28	0.132	0.192
99	RBM3	0.008	0.169
100	RBM30 (RBM4B)	0.094	0.335
101	RBM42	0.590	0.233
102	Rbm4a (RBM4)	0.074	0.102
103	RBM5	0.101	0.213
104	RBM6	0.127	0.184
105	RBM7	0.015	0.127
106	RBM8A	0.005	0.399
107	RBMX	0.022	0.116
108	RBMX2	0.007	0.296
109	RBPMS	0.561	0.216
110	RDBP (NELFE)	0.007	0.280
111	RIP-1 (KRR1)	0.035	0.255
112	RNPS1	0.023	0.274
113	Ro autoantigen 60 kDa (RO60)	Trace	0.119
114	RPP25	0.270	0.197
115	RUNX3	0.001	0.136
116	SAF-A (HNRNPU)	Undetectable	N/A

No	Protein/gene name (HGNC gene symbol)	Knockdown (KD) efficiency (Control KD=1)	PSI (control KD=0.103)
117	SAFB	0.115	0.221
118	Sam68 (KHDRBS1)	0.018	0.235
119	SART3	0.080	0.173
120	SC35 (SRSF2)	1.536	N/A
121	SERBP1	0.222	0.291
122	set1 (SETD1A)	0.230	0.248
123	SF2 (SRSF1)	0.011	0.164
124	SFRS10 (TRA2B)	0.377	0.249
**125**	**SPF45 (RBM17)**	**0.023**	**0.454**
126	SR140 (U2SURP)	0.234	0.250
127	SRM300 (SRRM2)	0.576	0.231
128	SRp20 (SRSF3)	0.040	0.222
129	SRp30c (SRSF9)	0.026	0.264
130	SRp38 (SRSF10)	0.290	0.204
131	SRp40 (SRSF5)	0.050	0.207
132	SRp54 (SRSF11)	0.119	0.274
133	SRp75 (SRSF4)	0.183	0.138
134	SRrp86 (SREK1)	0.019	0.102
135	STRBP	0.023	0.123
136	TAF15	0.264	0.158
137	TAP (NXF1)	0.219	0.099
138	TAT-SF1 (HTATSF1)	0.008	0.164
139	TEP1	Undetectable	N/A
140	TIA1	0.139	0.172
141	$U2AF^{35}$ (U2AF1)	0.031	0.113
142	$U2AF^{65}$ (U2AF2)	0.032	0.120
143	UBAP2L	0.018	0.125
144	vigilin (HDLBP)	0.023	0.102
145	vparp (PARP4)	0.041	0.375
146	WF9/2C7 (ZRSR2)	0.171	0.198
147	WF9/5A7 (RBM39)	0.093	0.133
148	wig-1 (ZMAT3)	1.312	N/A
149	YB-1 (YBX1)	0.015	0.171
150	ZC3H6	0.003	0.094
151	ZFR	0.079	0.119
152	ZNF335	0.007	0.114
153	ZNF74	0.243	0.213
154	9G8 (SRSF7)	2.200	N/A

Table S2 List of synthetic oligonucleotides used in the experiments

**siRNAs (sense sequences) for cellular knockdown (dT: deoxyribonucleotide)**		
SPF45-siRNA#1	5′-GAACAAGACAGACCGAGAUdTdT-3′	Knockdown of SPF45
SPF45-siRNA#2	5′-GACCCUAUGUUUCCUAAUGdTdT-3′	Knockdown of SPF45
$U2AF^{65}$-siRNA	5′-GCACGGUGGACUGAUUCGUdTdT-3′	Knockdown of $U2AF^{65}$
PRP43-siRNA	5′-AAACAGAAAUGCAGGAUAAdTdT-3′	Knockdown of PRP43
SF4-siRNA	[SMARTpool ON-TARGETplus Human SUGP1 siRNA (horizon)]	Knockdown of SF4
**Primer DNAs for plasmid constructions**		
HNRNPH1-E7F-EcoRI	5′-GGAATTCCGCTATGGAGGCTATGATGA-3′	Construction of pcDNA3-HNRNPH1
HNRNPH1-E8R-XhoI	5′-CCCTCGAGGGCAGTAGCTCTGTAAGGTAAT-3′	
AdMLF-BamHI	5′-CGGGATCCCGACTCTCTTCCGCATCGCTG-3′	Construction of pcDNA3-AdML
AdMLR-EcoRI	5′-GGAATTCCCGTTCGGAGGCCGACGGGTT-3′	
EML3-E17F-EcoRI	5′-GGAATTCCGTGGTTGGTTTTGGACACAG-3′	Construction of pcDNA3-EML3
EML3-E18R-XhoI	5′-CCCTCGAGGGCATACAGCGGCCAAAGCGGC-3′	
MUS81-E13F-EcoRI	5′-GGAATTCCCCCTGGGAACCCTGAATCAG-3′	Construction of pcDNA3-MUS81
MUS81-E14R-XhoI	5′-CCGCTCGAGGGTGCTGTATCGATCCACCA-3′	
HNRNPH1-5′SSAdML-S	5′-GGAAGGGGTGAGTTAAGAATTGAATTTCTC-3′	Construction of pcDNA3-HNRNPH1/5′SSAdML
HNRNPH1-5′SSAdML-AS	5′-CTTAACTCACCCCTTCCAAATCTATCTGAC-3′	
HNRNPH1-BranchAdML-S	5′-GAAGGAGTCATACACTCTTGTCCATCTAGA-3′	Construction of pcDNA3-HNRNPH1/BranchAdML & pcDNA3-HNRNPH1//AdML 5'SS-BP-3'SS
HNRNPH1-BranchAdML-AS	5′-AAGAGTGTATGACTCCTTCAACTGAGAAAT-3′	
HNRNPH1-3′SSAdML-S	5′-TCCATACAGCTCTCAATTACTGTTTTTCAG-3′	Construction of pcDNA3-HNRNPH1/3′SSAdML
HNRNPH1-3′SSAdML-AS	5′-ATTGAGAGCTGTATGGACAAGAGTGTAAGC-3′	
HNRNPH1-AdMLPPT25-S	5′-TTATCCTGTCCCTTTTTTTTCCACAGACCTCAATTACTGTTTTTC-3′	Construction of pcDNA3-HNRNPH1/AdML-PPT25
HNRNPH1-AdMLPPT25-AS	5′-GAAAAAAAAGGGACAGGATAAGTAAGCATCCTTCAACTGAG-3′	
HNRNPH1-AdMLPPT25mt-S	5′-TTATCCTGTCGCTGTTGTGTCCACAGACCTCAATTACTGTTTTTC-3′	Construction of pcDNA3-HNRNPH1/AdML-PPT25mt
HNRNPH1-AdMLPPT25mt-AS	5′-GACACAACAGCGACAGGATAAGTAAGCATCCTTCAACTGAG-3′	
HNRNPH1-AdMLPPT13-S	5′-CTTTTTTTTCCACAGACCTCAATTACTGTTTTTCAG-3′	Construction of pcDNA3-HNRNPH1/AdML-PPT13
HNRNPH1-AdMLPPT13-AS	5′- TGAGGTCTGTGGAAAAAAAAGTGTAAGCATCCTTCAACTG -3′	
HNRNPH1-5′AdML-S1	5′-AGATAGATTTGGAAGAGGTAAGGACTCCCTCTCAAAAGCGG-3′	Construction of pcDNA3-HNRNPH1/5′AdML
HNRNPH1-5′AdML-AS1	5′-GATGGACAAGAGTGTAAGCAATCATCAAGGAAACCCTGG-3′	
HNRNPH1-5′AdML-S2	5′-CCAGGGTTTCCTTGATGATTGCTTACACTCTTGTCCATC-3′	
HNRNPH1-5′AdML-AS2	5′-CCGCTTTTGAGAGGGAGTCCTTACCTCTTCCAAATCTATCT-3′	
HNRNPH1-XhoIx2-S	5′-TGCTTACACTCGAGCTCGAGCTCTTGTCCATCTAGACCTC-3′	Construction of pcDNA3-HNRNPH1/XhoI x2
HNRNPH1-XhoIx2-AS	5′-CTCGAGCTCGAGTGTAAGCATCCTTCAACTGAG-3′	
HNRNPH1-AdMLPPT13-XhoIx2-S	5′-CTCGAGCTCGAGCTTTTTTTTCCACAGACCTCAATTACTGTTTTTCAG-3′	Construction of pcDNA3-HNRNPH1/AdML-PPT13/XhoI x2
HNRNPH1-AdMLPPT13-XhoIx2-AS	5′-GAAAAAAAAGCTCGAGCTCGAGTGTAAGCATCCTTCAACTG-3′	
EML3-AdMLPPT25-S	5′-TTATCCTGTCCCTTTTTTTTCCACAGATGGGTTGTACCTGGCCAT-3′	Construction of pcDNA3-EML3/AdML-PPT25
EML3-AdMLPPT25-AS	5′-GAAAAAAAAGGGACAGGATAACTCAGAGAGGGAAGGGCCA-3′	
EML3-AdMLPPT13-S	5′-CTTTTTTTTCCACAGACCGGTTGTACCTGGCCATTG-3′	Construction of pcDNA3-EML3/AdML-PPT13
EML3-AdMLPPT13-AS	5′-GGTCTGTGGAAAAAAAAGCTCAGAGAGGGAAGGGCC-3′	
EML3-5′AdML-S1	5′-GTACAGCCCAGGTGGGAACTCCCTCTCAAAAGCGG-3′	Construction of pcDNA3-EML3/5′AdML
EML3-5′AdML-AS1	5′-GCAAATACAGTCACTCAGAGATCATCAAGGAAACCCTGG-3′	
EML3-5′AdML-S2	5′-CCAGGGTTTCCTTGATGATCTCTGAGTGACTGTATTTGC-3′	
EML3-5′AdML-AS2	5′-CCGCTTTTGAGAGGGAGTTCCCACCTGGGCTGTAC-3′	
SPF45-F-EcoRI	5′-GGAATTCTGATGTCCCTGTACGATGACCT-3′	Construction of pcDNA3-Flag/SPF45
SPF45-R-XhoI	5′-CCCTCGAGGGTCAAACTTGTTCTGCCAAATCC-3′	
SPF45-UHMmt-F	5′-GTGCGGGAGAGGTGAAGGAAGACTTGGAAG-3′	Construction of pcDNA3-Flag-SPF45/UHMmt
SPF45-UHMmt-R	5′-CACCTCTCCCGCACCAACCA-3′	

SPF45-ΔG-F	5′-CTTCCTCGCTGGCGACGCCACAGAGAAAGA-3′	Construction of pcDNA3-Flag-SPF45/ΔG
SPF45-ΔG-R	5′-TGGCGTCGCCAGCGAGGAAGGAGTTGCTAG-3′	
SPF45-siR-F	5′-CCAATGTTCCCAAACGATTATGAGAAAGTA-3′	Construction of pcDNA3-Flag-SPF45/siR, pcDNA3-Flag-SPF45/UHMmt/siR & pcDNA3-Flag-SPF45/ΔG/siR
SPF45-siR-R	5′-TTTGGGAACATTGGATCATATTCGTCAGCT-3′	
**Primer DNAs for analysis**		
HNRNPH1-E7F-EcoRI	See above	Splicing assay of endogenous HNRNPH1 intron 7
HNRNPH1-E8R-XhoI	See above	
RFC4-E9F-EcoRI	See above	Splicing assay of endogenous HNRNPH1 intron 7
RFC4-E10R-XhoI	See above	
EML3-E17F-EcoRI	See above	Splicing assay of endogenous EML3 intron 17
EML3-E17R-XhoI	See above	
DUSP1-F	5′-TGCAGTACCCCACTCTACGA-3′	Splicing assay of endogenous DUSP1 intron 2
DUSP1-R	5′-GAGACGTTGATCAAGGCAGTG-3′	
NFKBIA-F	5′- TCCTCAACTTCCAGAACAACC-3′	Splicing assay of endogenous NFKBIA intron 2
NFKBIA-R	5′-TCAGCAATTTCTGGCTGGT-3′	
MUS81-E13F-EcoRI	5′-GGAATTCCCCCTGGGAACCCTGAATCAG-3′	Splicing assay of endogenous MUS81 intron 13
MUS81-E14R-XhoI	5′-CCGCTCGAGGGTGCTGTATCGATCCACCA-3′	
RECQL4-E15F-EcoRI	5′-GGAATTCCAAGACCTGCGAGAGCTGCG-3′	Splicing assay of endogenous RECQL4 intron 15
RECQL4-E16R-XhoI	5′-CCGCTCGAGGCAGTTCAGACGGCAATGGG-3′	
MTA1-E5F-EcoRI	5′-GGAATTCCGAAATGGAGAACCCGGAAATG-3′	Splicing assay of endogenous MTA1 intron 5
MTA1-E6R-XhoI	5′-CCGCTCGAGGCTCCAGGTAGGACTTGAG-3′	
AdML-E1F	5′-CGTTCGTCCTCACTCTCTTCCGC-3′	Splicing assay of AdML mini-gene pre-mRNA (FIG. 3)
AdML-E2R	5′-ACCGCGAAGAGTTTGTCCTCAACC-3′	
T7	5′-AATACGACTCACTATAG-3′	Splicing assay of AdML mini-gene pre-mRNA (FIG. 6)
AdML-E2R	5′-ACCGCGAAGAGTTTGTCCTCAACC-3′	
T7	See above	Splicing assay of HNRNPH1 mini-gene pre-mRNA
HNRNPH1-E8R-XhoI	See above	
T7	See above	Splicing assay of EML3 mini-gene pre-mRNA
EML3-E17R-XhoI	See above	
T7	See above	Splicing assay of NUS81 mini-gene pre-mRNA
MUS81-E14R-XhoI	See above	
AdML-I1F	5′-GACTTCTGCGCTAAGATTGTCA-3′	qPCR detection of AdML pre-mRNA in CLIP assay
AdML-I1R	5′-TTGTCTTTTCTGACCAGATGGA-3′	
HNRNPH1-E7F-EcoRI	See above	qPCR detection of HNRNPH1 pre-mRNA in CLIP assay
HNRNPH1-I7R	5′-GGTCTAGATGGACAAGAGTGT-3′	
EML3-E17F-EcoRI	See above	qPCR detection of EML3 pre-mRNA in CLIP assay
EML3-I17-R	5′-GCATGTGAGTCCAGGGTT-3′	
MUS81-E13F-EcoRI	See above	qPCR for detection of EML3 pre-mRNA in CLIP assay
MUS81-I13R	5′-CAGGCCATGTCTGAGAAGCT-3′	
SP6	5′-ATTTAGGTGACACTAT-3′	Reverse transcription in CLIP assay

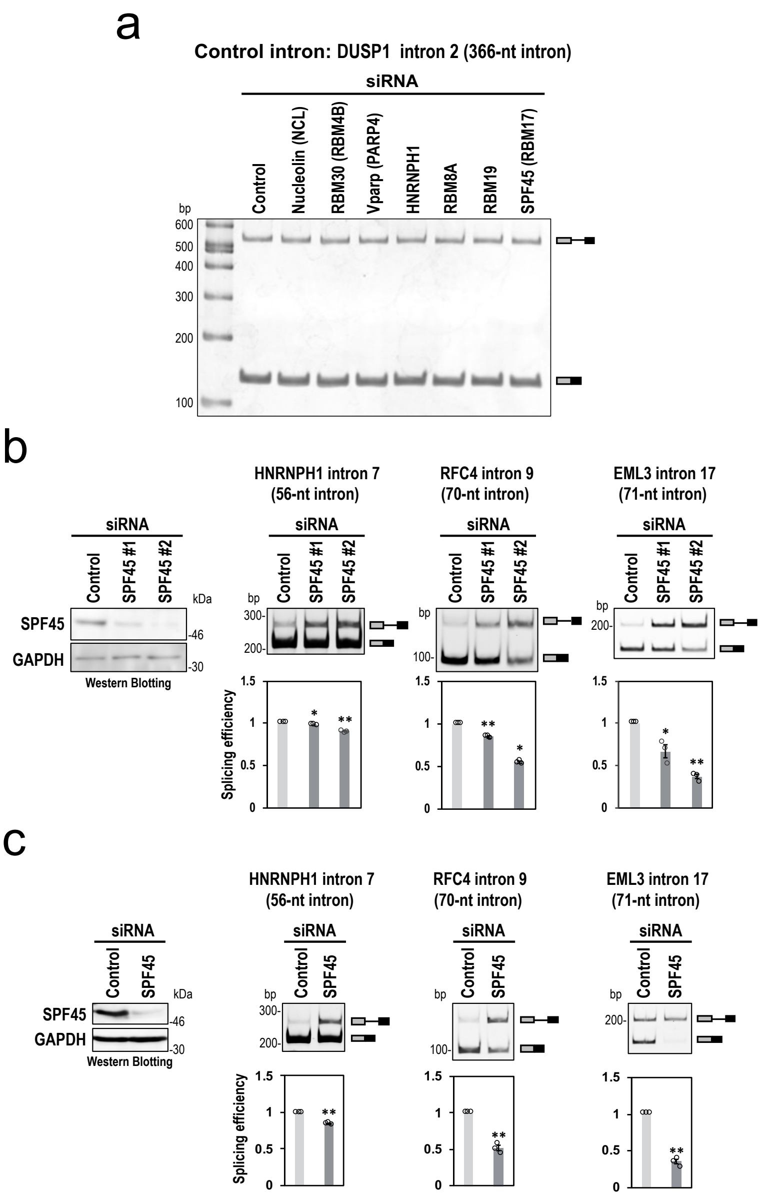

Fig. S1 Our discovered splicing in short introns is neither substrate-specific nor cell-specific events. **a,** The knockdown of top seven splicing repression factors, tested in the short HNRNPH1 intron (PSI>0.3, Table S1), do not affect conventional splicing in the control long intron. After the siRNA transfection in HeLa cells, endogenous splicing of the indicated control intron was analyzed by RT–PCR. Displayed PAGE gel is a representative of three independent experiments. **b,** Splicing repression in the three kinds of short introns is dependent on the knockdown efficiency. HeLa cells were transfected with control siRNA and two different siRNAs (#1, #2) targeting SPF45. The SPF45 protein depletion was checked by a Western blotting (left panel). *In cellulo* splicing assays of the indicated three short introns using RT–PCR. Means ± SEM are given for three independent experiments and two-tailed paired Student *t*-test values were calculated (HNRNPH1 intron: p=0.0104 for Control vs SPF45 #1 siRNA, p=0.0028 for Control vs SPF45 #2 siRNA; RFC4 intron: p=0.0037 for Control vs SPF45 #1 siRNA, p=0.0240 for Control vs SPF45 #2 siRNA; EML3 intron: p=0.0470 for Control vs SPF45 #1 siRNA, p=0.0017 for Control vs SPF45 #2 siRNA). $^{*}P < 0.05$, $^{**}P < 0.01$. **c,** Splicing repression in the three kinds of short introns is also observed in HEK293 cells. The same *in cellulo* splicing assays in panel 'b' were performed using SPF45-knockdown HEK293 cells (with siRNA #2). Means ± SEM are given for three independent experiments and two-tailed paired Student *t*-test values were calculated (HNRNPH1 intron: p=0.0015 for Control vs SPF45 siRNA #2; RFC4 intron: p=0.0058 for Control vs SPF45 siRNA #2; EML3 intron: p=0.0032 for Control vs SPF45 siRNA #2). $^{**}P < 0.01$. Source data of all the above panels are provided as a Source Data file.

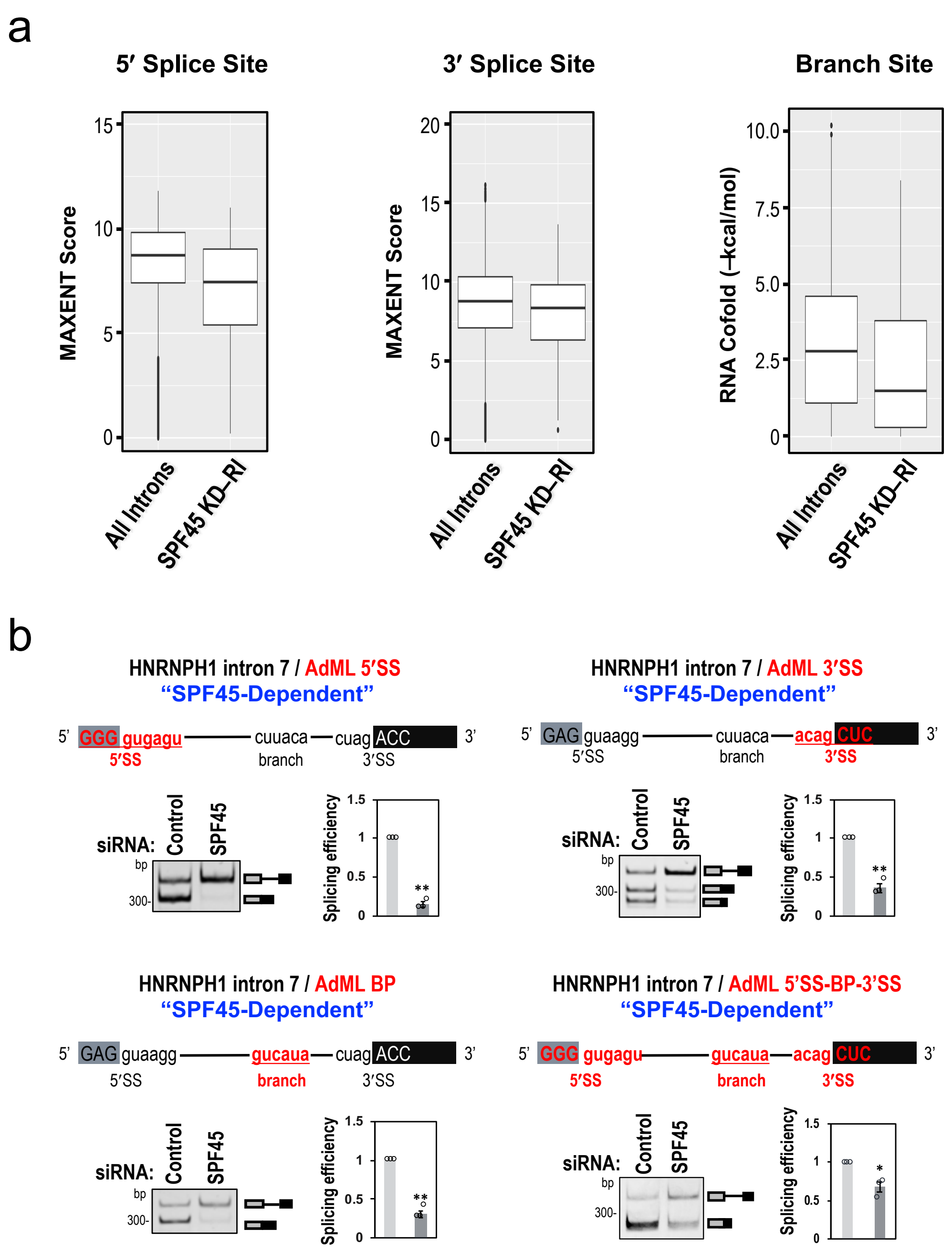

Fig. S2 Neither splice sites nor branch site is the determinant for SPF45-dependent splicing in short introns.
a, The box plots compare the strengths of the 5′/3′ splice sites and the branch site of all introns (in human RefGene) with those of the retained introns in SPF45-knockdown HEK293 cells. Box plots show the summary of the data set; median (middle line), 25–75th percentile (box), and <5th and >95th percentile (whiskers), together with outliers (single points). **b,** The splice sites and/or branch site sequences have no effect on SPF45-dependent splicing. Chimeric HNRNH1-intron 7 pre-mRNAs are schematically shown (red color indicates AdML derived sequences). These pre-mRNAs were expressed from mini-genes in HeLa cells and their splicing was assayed by RT–PCR. PAGE images and quantifications of RT–PCR are shown. Means ± SEM are given for three independent experiments and two-tailed paired Student *t*-test values were calculated (HNRNPH1 intron/AdML5′SS: p=0.0017 for Control vs SPF45 siRNA; HNRNPH1 intron/AdML BP: HNRNPH1 intron/AdML 5′SS-BP-3′SS: p=0.0415 for Control vs SPF45 siRNA). $*P < 0.05$, $**P < 0.01$. Source data of all the above panels are provided as a Source Data file.

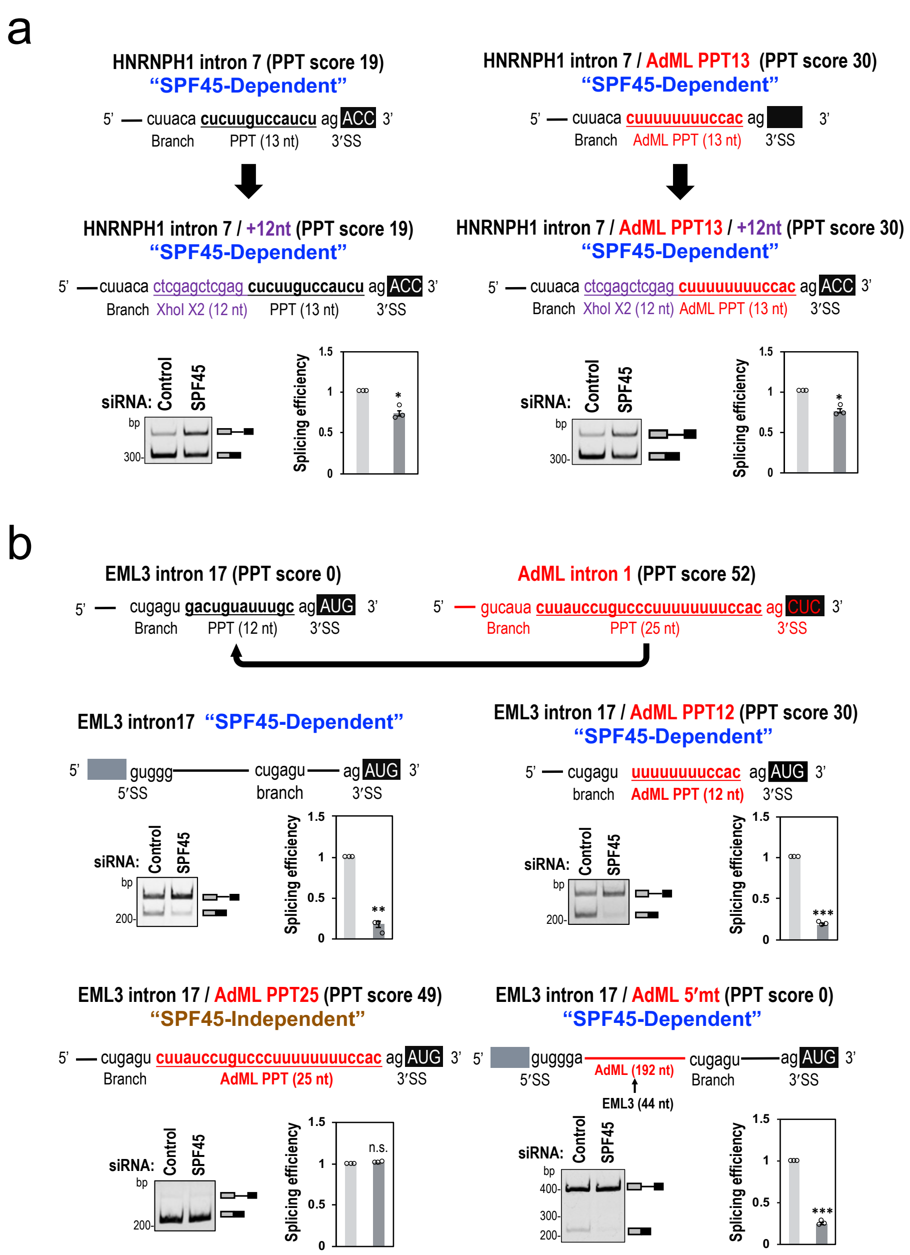

Fig. S3 Truncated polypyrimidine tract (PPT) *per se* is the determinant for SPF45-dependent splicing in short introns.
a, Extending the distance between the branch site and the 3′ splice site has no effect on SPF45-dependent splicing. Original HNRNPH1 pre-mRNA and variant pre-mRNAs with AdML PPT and extended 12-nt fragment (dimer of XhoI site) are schematically shown (red color: AdML derived sequences). Means ± SEM are given for three independent experiments and two-tailed paired Student *t*-test values were calculated (HNRNPH1 intron/+12nt: p=0.0305 for Control vs SPF45 siRNA; HNRNPH1 intron/AdML PPT13/+12nt: p=0.0214 for Control vs SPF45 siRNA). *P < 0.05. **b,** Critical role of the truncated PPT is also verified in the another SPF45-dependent short intron. Original EML3 and AdML pre-mRNAs, and chimeric EML3 pre-mRNAs are schematically shown (red color: AdML derived sequences). See Fig. 3 for the *in cellulo* splicing assays. Means ± SEM are given for three independent experiments and two-tailed paired Student *t*-test values were calculated (EML3 intron: p=0.0025 for Control vs SPF45 siRNA; EML3 intron/AdML PPT25 p=0.1190 for Control vs SPF45 siRNA; EML3 intron/AdML PPT12: p=0.0001 for Control vs SPF45 siRNA; EML3 intron/AdML 5'mt: p=0.0004 for Control vs SPF45 siRNA). **P < 0.01, ***P < 0.001, n.s.= not statistically significant P > 0.05. Source data of all the above panels are provided as a Source Data file.

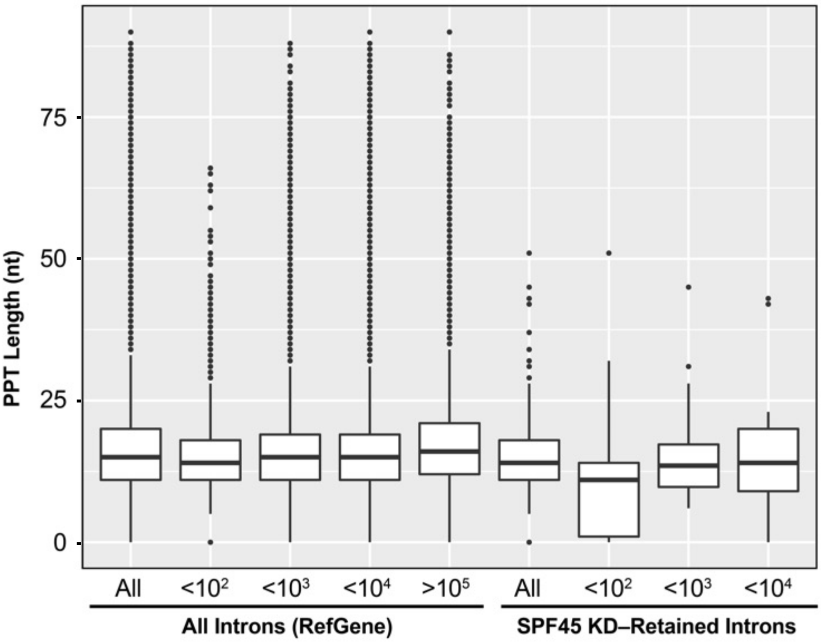

Fig. S4 Truncated polypyrimidine tract (PPT) is dominated in the set of the SPF45-dependent introns shorter than 100 nt.
The box plots compare the PPT-length distributions of all introns (in human RefGene) with those of the retained introns in SPF45-knockdown HEK293 cells. The numbers of introns are indicated in parentheses (discrepancy of the total numbers comparing with Fig. 2b is due to the elimination of introns with ambiguous branch point and PPT). Box plots show the summary of the data set; median (middle line), 25–75th percentile (box), and <5th and >95th percentile (whiskers), together with outliers (single points). Source data are provided as a Source Data file.

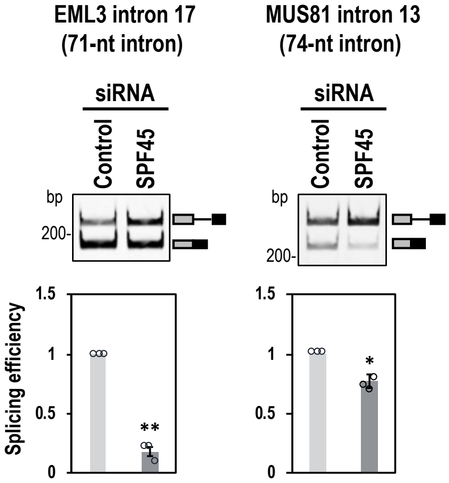

Fig. S5 The SPF45-dependency is recapitulated in ectopically expressed two mini-genes including short introns.
In cellulo splicing assays (see Fig. 3) of EML3-intron 17 and MUS81-intron 13 pre-mRNAs were performed using control siRNA- and SPF45 siRNA-treated HeLa cells (see Fig. 1 for the depletion efficiency of SPF45). Means ± SEM are given for three independent experiments and two-tailed paired Student *t*-test values were calculated (EML3 intron: p=0.0025 for Control vs SPF45 siRNA; MUS81 intron: p=0.0157 for Control vs SPF45 siRNA). $^*P < 0.05$, $^{**}P < 0.01$. Source data of the above panels are provided as a Source Data file.

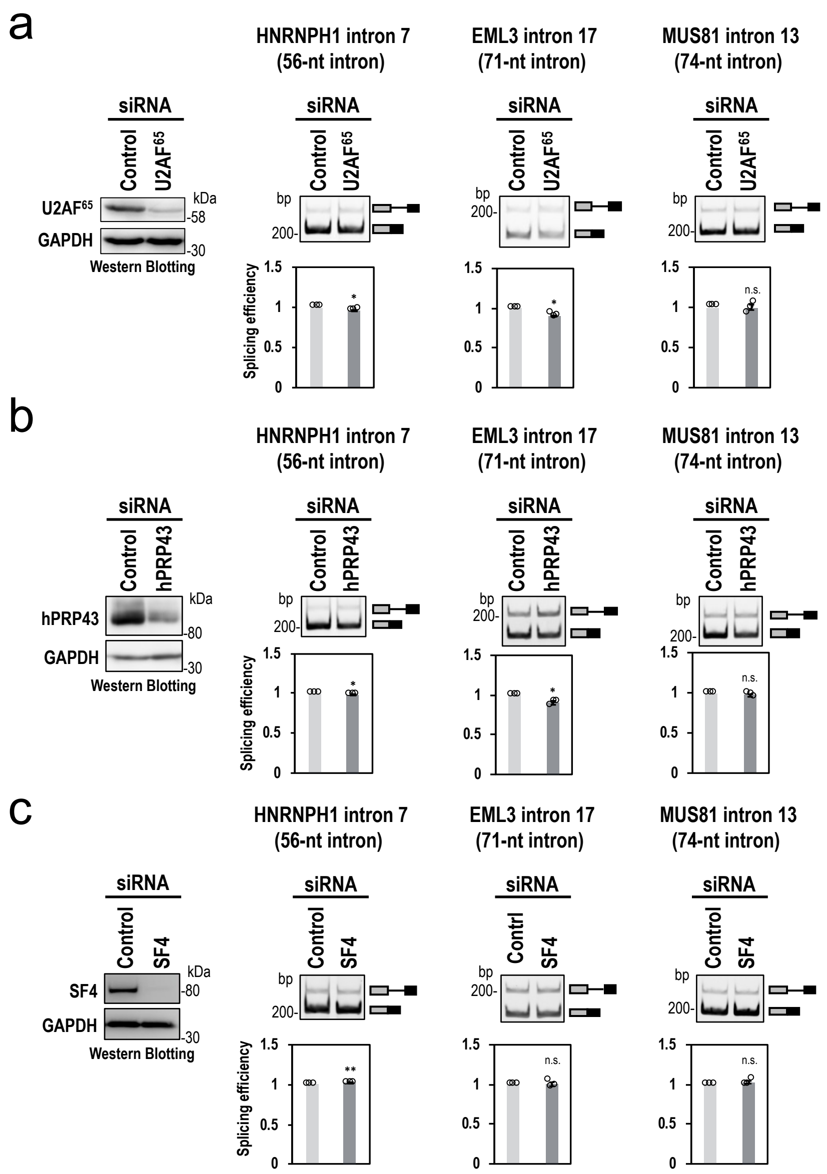

Fig. S6 Knockdown of the potential SPF45-interacting factors (U2AF^{65}, hPRP43 and SF4) has no effect on SPF-45 dependent splicing.
a, HeLa cells were transfected with control siRNA and siRNA targeting U2AF^{65}. At 72 h post-transfection, the U2AF^{65} protein depletion was checked by a Western blotting with indicated antibodies (left panel). *In cellulo* splicing assays of the indicated three short introns using RT–PCR (see also Fig. S1). Means ± SEM are given for three independent experiments and two-tailed paired Student *t*-test values were calculated (HNRNPH1 intron: p=0.0201 for Control vs U2AF^{65} siRNA; EML3 intron: p=0.0223 for Control vs U2AF^{65} siRNA; MUS81 intron: p=0.9882 for Control vs U2AF^{65} siRNA). *P < 0.05, n.s. *P > 0.05. **b,** HeLa cells were transfected with control siRNA (Ctl) and siRNA targeting hPRP43. See above for Western blotting and *in cellulo* splicing assays. Means ± SEM are given for three independent experiments and two-tailed Student *t*-test values were calculated (HNRNPH1 intron: p=0.0295 for Control vs hPRP43 siRNA; EML3 intron: p=0.0405 for Control vs hPRP43 siRNA; MUS81 intron: p=0.2182 for Control vs hPRP43 siRNA). *P < 0.05, n.s. *P > 0.05. **c,** HeLa cells were transfected with control siRNA (Ctl) and siRNA targeting SF4. See above for Western blotting and *in cellulo* splicing assays. Means ± SEM are given for three independent experiments and two-tailed paired Student *t*-test values were calculated (HNRNPH1 intron: p=0.0072 for Control vs SF4 siRNA; EML3 intron: p=0.8010 for Control vs SF4 siRNA; MUS81 intron: p=0.3989 for Control siRNA vs SF4 siRNA). **P < 0.01, n.s. *P > 0.05. Source data of all the above panels are provided as a Source Data file.

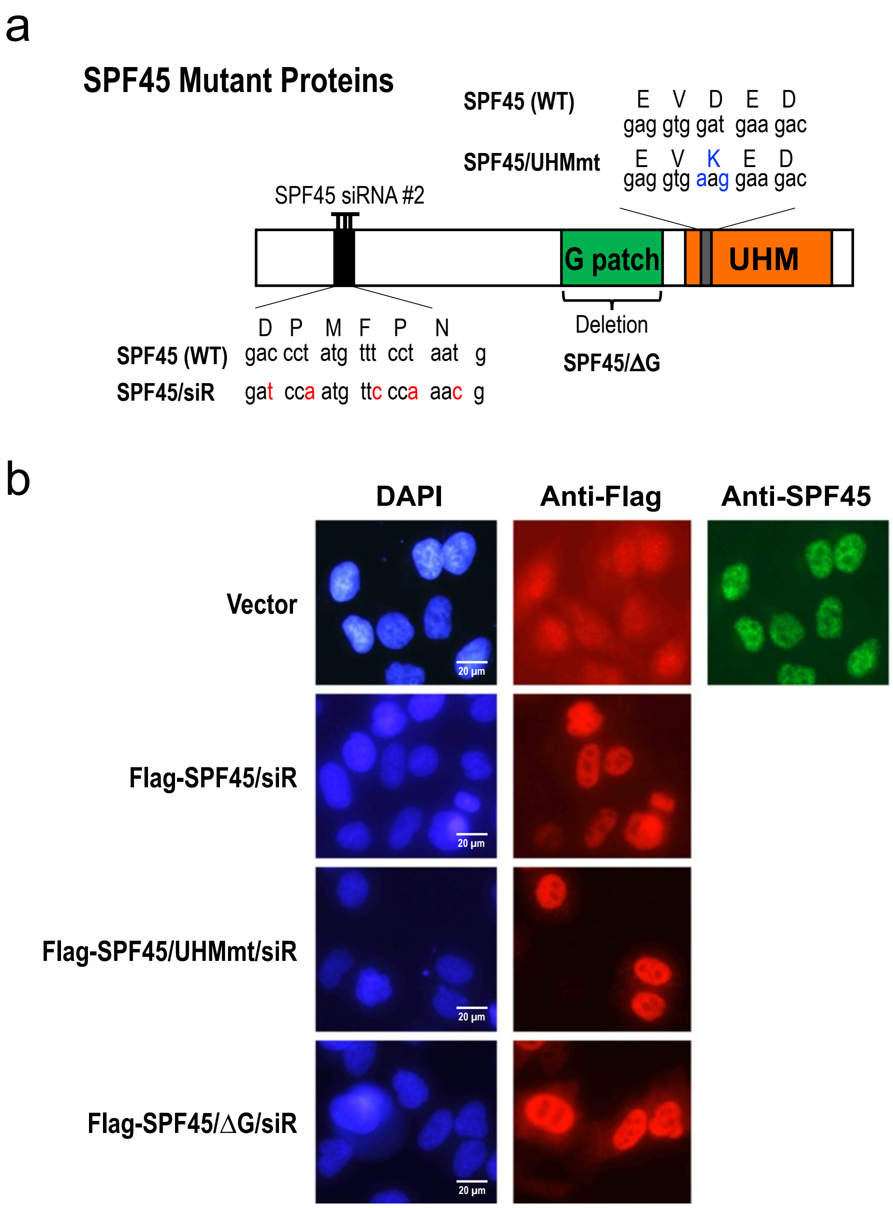

Fig. S7 Subcellular localization of SPF45/siR, SPF45/UHMmt/siR, and SPF45/ΔG/siR proteins.
a, Schematic structures of SPF45 and the induced mutations in the indicated expression plasmids. The D→K mutation in SPF45/UHMmt, whose protein cannot binds ULM, is highlighted in blue. The target of SPF45-siRNA#2 is depicted and introduced five silent siRNA-resistant mutations were highlighted in red.
b, HeLa cells were transfected with Flag-SPF45/siR, Flag-SPF45/UHMmt/siR, and Flag-SPF45/ΔG/siR expression plasmids and their subcellular localizations were examined by immunofluorescence microscopy using indicated antibodies. Cells were also stained with DAPI to detect nuclei. Immunofluorescence microscopic assays are representative of three independent experiments. Source data of the above microscopy images are provided as a Source Data file.

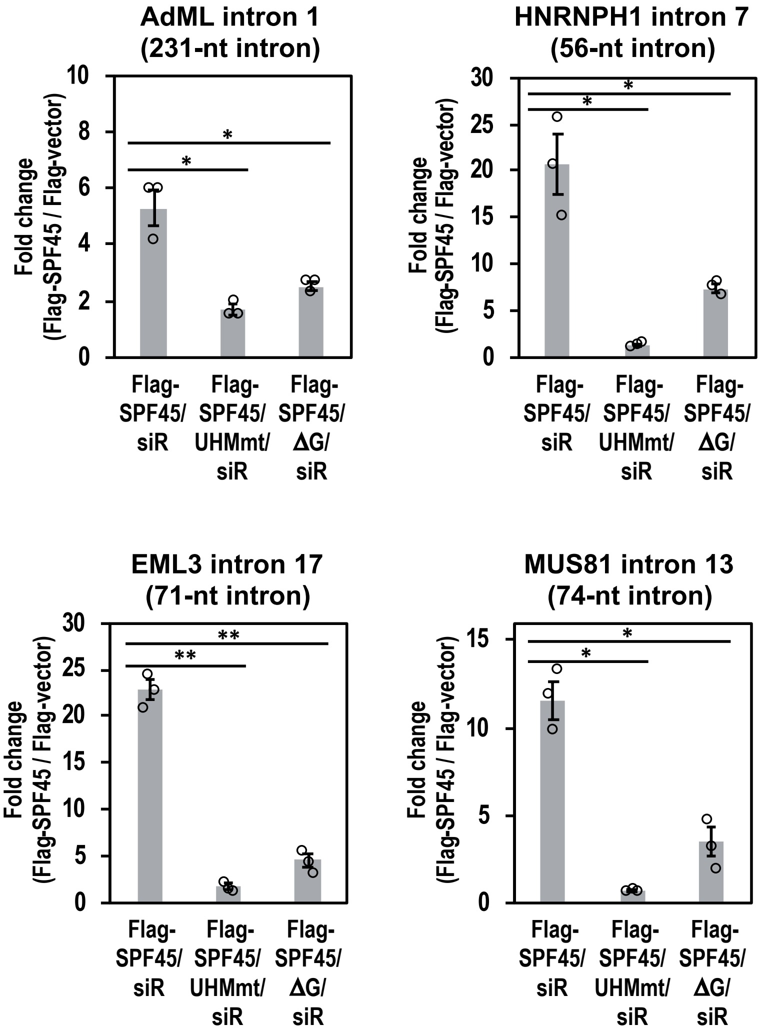

FIG. S8 Specific association of SPF45 to short introns is dependent on the binding to SF3b155 through UHM–ULM interaction.
In cellulo formaldehyde crosslinking and immunoprecipitation analysis (see Fig. 4a) demonstrates that the SPF45 association to indicated three short introns is drastically impaired by the UHM mutation in SPF45. Since SPF45 can bind to both short and control introns *via* five ULMs in SF3b155 (see Fig. 4a), it is consistent to observe the impairment also in control AdML intron. Means ± SEM are given for three independent experiments and two-tailed paired Student *t*-test values were calculated (AdML intron: p=0.0470 for Flag-SPF45/siR vs Flag-SPF45/UHMmt/siR; AdML intron: p=0.0277 for Flag-SPF45/siR vs Flag-SPF45/ΔG/siR; HNRNPH1 intron: p=0.0270 for Flag-SPF45/siR vs Flag-SPF45/UHMmt/siR; HNRNPH1 intron: p=0.0394 for Flag-SPF45/siR vs Flag-SPF45/ΔG/siR; EML3 intron: p=0.0041 for Flag-SPF45/siR vs Flag-SPF45/UHMmt/siR; EML3 intron: p=0.0072 for Flag-SPF45/siR vs Flag-SPF45/ΔG/siR; MUS81 intron: p=0.0101 for Flag-SPF45/siR vs Flag-SPF45/UHMmt/siR; MUS81 intron: p=0.0358 for Flag-SPF45/siR vs Flag-SPF45/ΔG/siR). *P < 0.05, **P < 0.01. Source data of the above graphs are provided as a Source Data file.

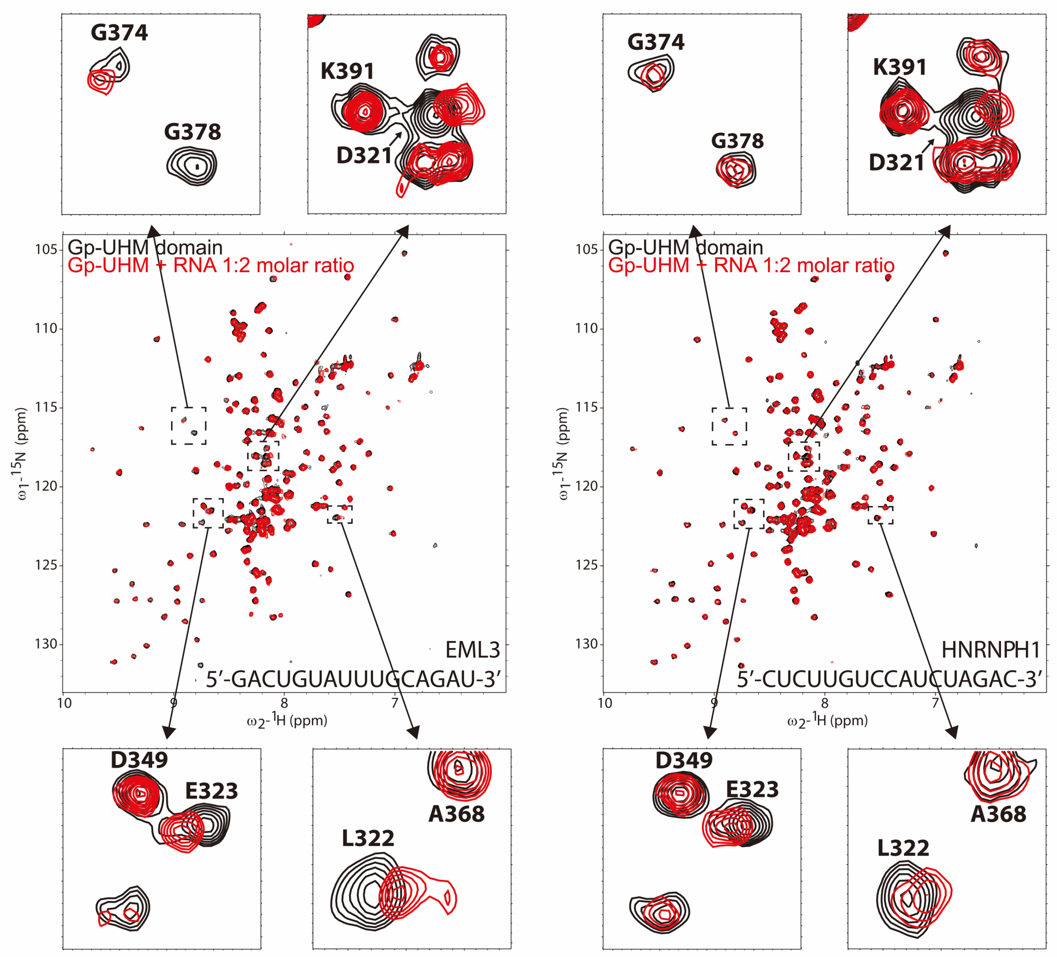

FIG. S9 The G-patch motif and UHM domain of SPF45 do not bind significantly to RNA sequences of the truncated PPT from two short introns.
Comparison of NMR ^{1}H,^{15}N correlation spectra of SPF45 G-patch–UHM domain after 2-molar excess addition of the truncated PPTs from EML3 (left panel) and hnRNPH1 (right panel). Only minor changes (small chemical shift changes and line-broadening) are observed, which suggest non-specific and very weak interactions (with high micro-molar K_D).
