# Supplementary material for: SPF45/RBM17-dependent, but not U2AF-dependent, splicing in a distinct subset of human short introns
Source: Nat Commun. 2021 Aug 13;12:4910. doi: 10.1038/s41467-021-24879-y (PMC8363638; doi:10.1038/s41467-021-24879-y)
Supplement: Supplementary file 4 — Description of additional supplementary files [file 41467_2021_24879_MOESM4_ESM.docx]

Description of additional supplementary information

Title: supplementary dataset 1

Description: The list of retained introns in SPF45-knockdown HEK293 cells.
